# Uterine Dehiscence: A Rare Cause of Postpartum Puerperal Sepsis

**DOI:** 10.7759/cureus.18264

**Published:** 2021-09-25

**Authors:** Manjunath Haridas, Venkata Jaya Divya Tenneti, Amey Joshi

**Affiliations:** 1 Surgery, Manipal Hospital, Bangalore, IND; 2 General Surgery, Manipal Hospital, Bangalore, IND

**Keywords:** postpartum, myometrial closure, c-section, uterine dehiscence, intra-abdominal infection

## Abstract

Uterine dehiscence (partial or complete) is a rare complication of lower segment cesarean section (LSCS). Puerperal sepsis with intra-abdominal abscess following this event has been rarely reported. The delay in diagnosis and management of the condition can result in significant morbidity and mortality. We herein report three cases of puerperal sepsis along with intra-abdominal abscess associated with uterine dehiscence following LSCS. These patients in the current case series presented with complaints of fever and abdominal pain. Early recognition and prompt treatment with diagnostic laparoscopy and or laparotomy with drainage were effective in the management of these patients.

## Introduction

Uterine dehiscence (UD) due to endomyometritis (puerperal sepsis) in the postpartum period, following lower segment cesarean section (LSCS) delivery, is an infrequent occurrence with limited literature [1]. The weakening of uterine closure/scar tissue due to infection and subsequent spillage of pathogenic organisms into the peritoneum may result in peritonitis or abscess formation [2,3]. Similar outcomes have been observed in cases of faulty approximation of the myometrium during LSCS, which allowed the gradual spread of intra-uterine pathological organisms into the peritoneal cavity [2,4]. 

Here, we report the presentation and successful management of three cases of puerperal sepsis following uterine dehiscence (partial or complete), which were complicated with the formation of intra-abdominal abscesses.

## Case presentation

Case 1

**Figure 1:**
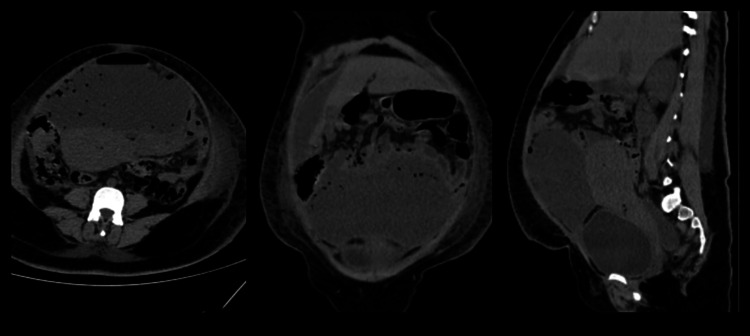
CT scan showing localized fluid collection anterior to the uterus, beneath the anterior abdominal wall, and superior to the bladder extending to the flanks with wall enhancement, highly suggestive of intra-abdominal abscess.

A 28-year-old primigravida with an insignificant past medical and surgical history underwent elective LSCS. The surgery was uneventful and delivered a healthy full-term boy. Three days following the surgery, the patient noted intermittently spiking fevers along with diffuse abdominal pain. At an outpatient postnatal visit, she was prescribed antipyretics and antibiotics for three days. However, due to worsening of symptoms, she was transferred to our tertiary care hospital on day eleven of puerperium. The patient was tachycardic, tachypneic, febrile (100°F), and hypoxic. A moderate amount of purulent discharge was noted at her surgical site. Emergency computed tomography (CT) of the abdomen revealed a localized fluid collection anterior to the uterus, beneath the anterior abdominal wall, and superior to the bladder extending to the flanks with wall enhancement, highly suggestive of an intra-abdominal abscess (Figure 1). She underwent an emergency exploratory laparotomy. Frank pus collection was noted in the paracolic, subphrenic, sub-hepatic, paravesicular regions and in between bowel loops. Eight hundred milliliter of the pus was drained and sent for culture and sensitivity. Complete dehiscence of the uterine closure in the lower-segment incision site was also noted. Suture material was removed, and the incision margin was freshened. The uterine cavity was normal, with no evidence of pus and active bleeding. Re-suturing of the uterine incision was done.

The small bowel was unremarkable for serosal abrasions or perforations. Two intra-abdominal drains were left in situ. Postoperatively, the patient was kept on lung-protective ventilation with noradrenaline support. On postoperative day two, she was extubated and weaned off vasopressor support. She was hemodynamically stable and was tolerating small oral feeds. Postoperative day four was significant for fever spikes with increased white blood cell count (24,000/cumm) requiring antibiotics as per pus culture sensitivity reports. The patient improved symptomatically with a decrease in white blood cell (WBC) counts and was discharged on hospital day seven.

Case 2

A 31-year-old primigravida with an unremarkable antenatal history had delivered a full-term baby by an uncomplicated elective LSCS at an outside facility. She initially complained of lower abdominal pain which was managed conservatively. On day 11, her symptoms worsened which brought her to our emergency room at our tertiary care facility for further evaluation. 

**Figure 2:**
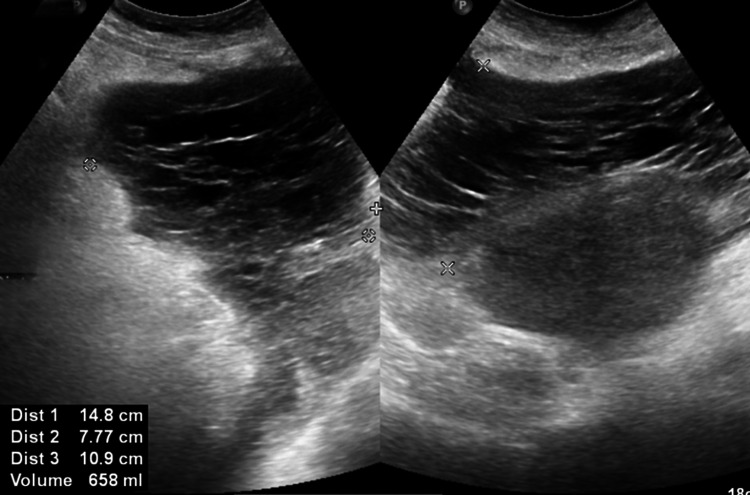
Ultrasound showing pus-filled lesion in the lower abdomen, anterior to the uterus and beneath the abdominal wall.

**Figure 3:**
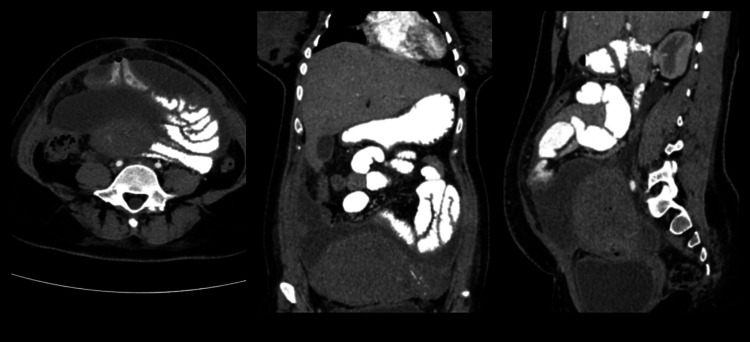
CT scan showing intra-abdominal fluid-filled lesion in the lower abdomen, anterior to the uterus and beneath the abdominal wall.

Physical examination was significant for tachycardia, tachypnoea, and fever (102°F). Abdominal examination revealed a tender lower abdomen and a tender surgical LSCS scar. Laboratory investigations were significant for leucocytosis (WBCs). Ultrasound and emergency CT scan of the abdomen revealed a pus-filled lesion in the lower abdomen, anterior to the uterus and beneath the abdominal wall (Figures 2, 3). The suspected abdominal abscess was confirmed with diagnostic laparoscopy and an emergency laparotomy was done in view of a well-formed abscess. The abdomen was opened through a midline vertical and previous Pfannenstiel incision. On evaluation, a dense loculated purulent fluid was adherent to the anterior abdominal wall and the anterior aspect of the uterus and bowel loops. This was excised and sent for culture and sensitivity. Systematic large and small bowel inspections did not reveal any perforation. The uterine incision site inspection revealed loosely approximated sutured margins of the myometrium along with a rent of 3 x 2 cm (complete uterine dehiscence). The uterine edges were debrided and re-sutured. The pouch of Douglas was explored, and inflammatory exudate was drained. The abdomen was closed in layers, and the skin was closed with staples. The patient withstood the procedure well. Postoperative day two revealed declining WBC counts, and the patient was doing symptomatically better.

Case 3

**Figure 4:**
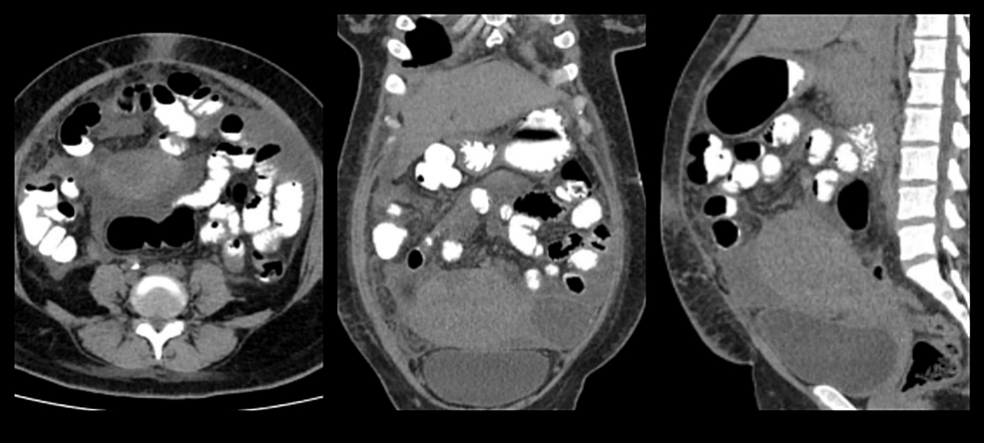
CT scan showing bulky postpartum uterus and moderate fluid collection with peritoneal thickening and enhancement with omental fat stranding.

A 37-year-old woman (gravida 3, aborta 2) delivered a healthy full-term baby by an uncomplicated LSCS. Past medical history was significant for well-controlled type 2 diabetes mellitus and hypothyroidism. Four days following LSCS, the patient developed abdominal distension, shortness of breath which was managed on an outpatient basis by her obstetrician and cardiologist. She later presented with intermittent fever which gradually worsened and brought her to our emergency room on day 10 of puerperium. Her WBC counts and C-reactive protein were elevated (21,900/cumm and 481, respectively). Ultrasonography of the abdomen revealed peritoneal collection with septations, and the patient was admitted for further management. An emergent CT scan of the abdomen revealed moderate fluid collection with peritoneal thickening and enhancement with omental fat stranding (Figure 4). The patient was started on antibiotics. She underwent an ultrasound-guided aspiration of the fluid, which on analysis, revealed staphylococcal growth. Based on culture sensitivity reports, antibiotics were changed. Due to persisting symptoms and an upward trend of WBC count (23,000/cumm), the patient underwent a diagnostic laparoscopy which revealed a well-formed pelvic abscess following which laparotomy was performed. The nidus of infection was drained which probably had the uterine source. A thickened omentum which was densely adherent to the uterine scar was noted. Two intra-abdominal drains were kept. Culture reports of the fluid showed growth of methicillin-susceptible *Staphylococcus aureus* (MSSA), and the patient was started on appropriate antibiotics. WBCs levels decreased, and the patient showed a significant clinical improvement. The patient improved symptomatically and hence was discharged.

## Discussion

We report the presentation and successful management of three cases of puerperal sepsis following uterine dehiscence (partial or complete), which were complicated with the formation of intra-abdominal abscesses. Our report highlights the rare occurrence of uterine dehiscence which may be secondary to improper closure techniques/suture materials. 

The worldwide increase in rates of cesarean section has linearly escalated the complications associated with it. Complications ranging from mild puerperal infections to endometritis, wound disruption, thrombophlebitis, chronic pelvic pain, adhesions, uterine scar dehiscence, and placental anomalies have been widely reported [2,5]. Postpartum uterine dehiscence, a rare clinical condition, is characterized by the opening of the incision line after cesarean section. Its incidence is 0.2-1.5% following a low transverse incision and about 4-9% following a classical incision [5]. Risk factors include diabetes, emergency surgery, closure technique, puerperal infection, and retrovesical or incision site hematoma [6]. The condition's pathophysiology primarily starts with a severe infection in the endometrial and myometrial layers of the uterus. This leads to necrosis of the weakest part of the uterine wall, typically the cesarean incision, thereby leading to its dehiscence [7]. Another hypothesis proposed includes the ischemic necrosis of myometrium due to improper closure techniques like tight locking sutures [4]. Symptoms of early uterine dehiscence range from heavy bleeding in the postpartum period to mild pelvic pain and suprapubic sensitivity [5]. With the spread of infection to the peritoneal cavity, symptoms of late uterine dehiscence like sepsis and anemia, along with clinical signs like fever, tachycardia, suprapubic tenderness, and per vaginal tenderness are invariably present [7]. 

Following uterine dehiscence, the uterine cavity lies in direct communication with the abdominal cavity. This allows pathogenic microorganisms from the uterus, which may have ascended via the genital tract, into the peritoneum, leading to peritonitis and sepsis. A combination of *Escherichia coli*, *Klebsiella pneumonia*, Streptococcus spp., and *Bacteroides fragilis*, with other Gram-negative, Gram-positive, and anaerobic bacteria are among the commonly cited organisms in postcesarean peritonitis [8].

One of the most dreaded complications of uterine dehiscence is a pelvic abscess, a localized collection of infected fluid in the pouch of Douglas, fallopian tube, ovary, or parametrial tissue, which usually starts as an ascending infection from the vagina [9]. However, in postoperative patients, blood loss, serous fluid, lymphatic debris, necrotic tissues accumulate in the lower pelvic area, and vaginal vault resulting in the formation of a simple fluid collection which later serves as a nidus of infection via skin contamination and vaginal opening and results in pelvic abscess formation [9,7].

Prompt recognition and diagnosis of pelvic abscess using imaging techniques such as ultrasonography, computed tomography, and magnetic resonance imaging are vital to prevent morbidity and mortality associated with it [5]. CT scan with oral contrast opacifies the bowel loop, and intravenous (IV) contrast enhances vascularity of the mass and opacifies the urinary tract. These amplify the diagnostic accuracy of the CT scan. The pelvic abscess presents as a hypodense collection with peripheral round or oval intensification on CT scan [10-12]. Despite these diagnostic tools, laparoscopy or exploratory laparotomy is considered the foremost for diagnosing and treating uterine scar dehiscence and repair [5]. During laparoscopy/exploratory laparotomy, pelvic abscess can be drained, along with evaluation for the nidus of infection, small bowel injury, uterine scar dehiscence can be assessed.

Alteration in the surgical technique with the closure of the uterine incision in two layers has significantly lowered the frequency of scar dehiscence. Avoidance of tight interlocking sutures leading to ischemic necrosis should be avoided [2]. The closure of the lower half of the subcutaneous layer with either interrupted or continuous sutures if the thickness of the abdominal wall exceeds 2 cm has been shown to decrease the risk of wound seromas significantly and wound disruptions by about 32% [13]. Since proper myometrial closure is propounded to play a role in the pathophysiology of uterine dehiscence, achieving an excellent myometrial approximation without ischemia is vital [4].

## Conclusions

Prompt recognition and evaluation of early postpartum abdominal pain and fever are of vital importance in preventing morbidity and mortality associated with puerperal sepsis. Imaging modalities like ultrasound and CT scans play a crucial role in the evaluation of patients presenting with severe sepsis. Early diagnostic laparoscopy and or laparotomy is recommended for source control. The use of proper surgical closure techniques including the use of appropriate suture material along with establishing a well-fitting uterine closure is vital. Following the standard guidelines for closure of uterine incisional layers, especially the myometrium and peritoneum are prime to prevent a life-threatening complication from an atypical seed of uterine dehiscence. 
